# A Case of Extensive Fracture and Depression of the Skull, in Which the Patient Recovered without an Operation

**Published:** 1844-08

**Authors:** D. C. Smyley

**Affiliations:** Pleasant Hill, Alabama


Art. III.—*A Case of Extensive Fracture and Depression of the Skull, in which the patient recovered without an operation.* By D. C. Smyley, M.D., of Pleasant Hill, Ala.

As I rode by the plantation of Mr. James Lawson, on the 24th of January last, I observed a mulatto girl, aged not quite 8 years, who had received, a moment before, a severe wound on her head from the falling limb of a tree. She was lying on the ground, apparently dead, the heart beating feebly at intervals. On examination, I found the left half of the frontal bone, except a small part which aids in forming the external angle of the eye, driven in upon the brain, the superciliary ridge pressing down immediately over the eye; the upper portion of the left parietal bone was also fractured and depressed, the fracture, about three inches in width, taking the sagittal suture as its upper line, and extending over a part of the accipital bone. My wrist, I believe, might have been buried in the fracture.

The first step was to elevate the superciliary ridge, and restore it to its place. This being done, the patient, for the first time, was seen to breathe. She was then taken to the house where dry clothing was provided. Her head, in the next place, was shaved, and reaction having come on to some extent, the depression began to disappear, the contents of the skull forcing out the fragments of bone which were greatly comminuted. Sinapisms were applied to her extremities which remained very cold. Her pulse was irregular in volume and beat. I attempted to give a dose of extract of elaterium, but found the patient was unable to swallow.

25th. Patient lay in a stupor all night; extremities still cold; considerable vomiting has taken place, with a large discharge of bile; pulse still irregular, and about 87 in the minute. To-day she is able to swallow. Gave three small doses of calomel and Dover's powder in the evening, and ordered an injection during the night.

26th. Rested badly last night; pulse 90, but not quite so irregular. Had two alvine evacuations early this morning, which were decidedly bilious. For the first time had a discharge of urine. Extremities have become warm. Before I arrived this morning half an ounce of blood had been taken, by which I was informed rigors were near being brought on. In the evening small portions of morphine and muriate of ammonia were given, which prescription was continued regularly throughout the treatment.

27th. Rested a little last night; skin quite pleasant; pulse 96, and becoming more regular. The head has become much swollen and puffed out, except immediately over the superior posterior angle of the parietal bone. An operation being proposed at this stage of the case, I objected on the ground that the patient seemed improved, and that the longitudinal sinus was probably lacerated. It was however agreed that it should be performed provided there was not a decided improvement by evening. Meantime, an injection was ordered. In the evening the patient began to evince some return of intellect. Directed two small doses of calomel and Dover's powder during the night.

28th. Part of the night patient rested well; had one evacuation early this morning; pulse more natural, but still 96. In the evening gave a dose of Epsom salts.

29th. Patient rested very well; had two stools during the night; pulse almost regular at 93. In the evening the swelling and ecchymosis which had extended to both eyes began to abate; ordered nourishment in the form of cornmeal gruel.

The patient continued to improve up to the 4th of February when she was dismissed cured. For six or eight weeks after this time she could not articulate distinctly, and even now cannot pronounce some words intelligibly.

On the day after the accident the wound was not much swollen, and on the slightest pressure with the finger over the fracture a crepitus was distinctly heard as between small pieces of bone. Not the slightest wound was inflicted upon the external teguments. The head on the injured side remains considerably flattened, and can never regain its original form. The superciliary arch of the left eye is also somewhat depressed. For about fourteen days incomplete paralysis of the right side existed, but has now disappeared. The mind appears clear, but the girl does not answer questions with the same promptness that was natural to her before the accident, and with this there is noticeable some languor in her bodily movements.

May 26, 1844.

